# Retina specific GCAPs in zebrafish acquire functional selectivity in Ca^2+^-sensing by myristoylation and Mg^2+^-binding

**DOI:** 10.1038/srep11228

**Published:** 2015-06-10

**Authors:** Stefan Sulmann, Farina Vocke, Alexander Scholten, Karl-Wilhelm Koch

**Affiliations:** 1 Department of Neurosciences, Biochemistry Group, University of Oldenburg, D-26111-Oldenburg, Germany

## Abstract

Zebrafish photoreceptor cells express six guanylate cyclase-activating proteins (zGCAPs) that share a high degree of amino acid sequence homology, but differ in Ca^2+^-binding properties, Ca^2+^-sensitive target regulation and spatial-temporal expression profiles. We here study a general problem in cellular Ca^2+^-sensing, namely how similar Ca^2+^-binding proteins achieve functional selectivity to control finely adjusted cellular responses. We investigated two parameters of critical importance for the trigger and switch function of guanylate cyclase-activating proteins: the myristoylation status and the occupation of Ca^2+^-binding sites with Mg^2+^. All zGCAPs can be myristoylated in living cells using click chemistry. Myristoylation does not facilitate membrane binding of zGCAPs, but it significantly modified the regulatory properties of zGCAP2 and zGCAP5. We further determined for all zGCAPs at least two binding sites exhibiting high affinities for Ca^2+^ with K_D_ values in the submicromolar range, whereas for other zGCAPs (except zGCAP3) the affinity of the third binding site was in the micromolar range. Mg^2+^ either occupied the low affinity Ca^2+^-binding site or it shifted the affinities for Ca^2+^-binding. Hydrodynamic properties of zGCAPs are more influenced by Ca^2+^ than by Mg^2+^, although to a different extent for each zGCAP. Posttranslational modification and competing ion-binding can tailor the properties of similar Ca^2+^-sensors.

Calcium sensor proteins mediate signaling processes that respond to changing concentrations of Ca^2+^-ions^1^,^2^. The binding of Ca^2+^ to intracellular calcium sensor proteins can trigger conformational transitions, which constitute a crucial step to regulate further downstream signaling proteins. One family of Ca^2+^-binding proteins named neuronal calcium sensor (NCS) proteins are predominantly expressed in neuronal tissue and are involved in diverse intracellular processes^2^,^3^. All NCS proteins harbor four EF-hand Ca^2+^-binding motifs, of which in most cases three (sometimes only two) motifs can bind micromolar to submicromolar Ca^2+^. One group of the NCS proteins is expressed in sensory cells and among them the guanylate cyclase-activating proteins (GCAPs) perform an important function in controlling the membrane bound guanylate cyclases (GCs) in retinal rod and cone cells^4^,^5^,^6^.

In their Ca^2+^-free, Mg^2+^-bound form GCAPs activate GCs, but they switch to an inhibitory mode, when all Ca^2+^-binding sites are filled with Ca^2+^ 7,8. Changing levels of cytoplasmic Ca^2+^ in rod and cone outer segments are linked to changing levels of the intracellular messenger cGMP. After light activation of the photoreceptor cell the intracellular cGMP level is depleted, leading to a shutdown of cyclic nucleotide gated (CNG) channels in the outer segment of the cell. This stops the influx of Ca^2+^, which is however still extruded by the continuous operation of a Na^+^/ Ca^2+^, K^+^ exchanger leading to a net decrease of cytoplasmic Ca^2+^. This decrease is sensed by GCAPs which in turn increase the GC activity, leading to re-opening of the CNG-channels and is a necessary step for the recovery of the photoreceptor to the dark-adapted state^4^,^5^,^6^,^7^,^8^,^9^.

Bovine and mice photoreceptor cells express two GCAP forms, GCAP1 and GCAP2, which bind to distant regions in the target GC and have different properties with respect to Ca^2+^-sensitivity, impact on catalytic efficiency of the target GC and different structural implications of the N-terminally attached myristoyl group^10^,^11^. Both GCAPs activate outer segment GCs in a Ca^2+^-relay mode fashion, where GCAP1 is activated at higher free Ca^2+^, followed by GCAP2, which becomes active, when Ca^2+^-levels have fallen to lower levels^9^,^10^,^11^. This Ca^2+^-relay system seems also to work in zebrafish rod and cone cells, where, however, the system is more complex due to the larger number of GCAP forms^12^. Zebrafish photoreceptor cells express a total of six GCAPs (zGCAP1, 2, 3, 4, 5 and 7)^13^,^14^ that differ in Ca^2+^-binding properties, Ca^2+^-sensitive GC regulation and spatial-temporal transcription/ expression profiles. Four zGCAPs, namely isoforms 3, 4, 5 and 7 are cone specific^12^,^14^,^15^.

Two parameters are of critical importance for the trigger and switch function of NCS proteins in general and GCAPs in particular: the myristoylation status and the occupation of EF-hand Ca^2+^-binding sites with Mg^2+^ 7,8,10,16,17. We have previously shown that zGCAP3 and 4 are myristoylated, when co-expressed with yeast *N*-myristoyltransferase (NMT) in *E.coli*^15^,^18^. Furthermore, zebrafish NMT is expressed in the developing larval retina at 5 days post fertilization (dpf) and is active as shown by modification of zGCAP3 after 7 dpf. In the present study we investigated in a comparative manner, whether all zGCAPs can be myristoylated in living cells using an approach of *in vivo* and *in vitro* click chemistry in combination with fluorescence microscopy. Revealing that all zGCAPs can exist in a myristoylated and non-myristoylated form we investigated its impact for target regulation and Ca^2+^-dependent membrane interaction. We further asked whether the presence of physiological Mg^2+^ can influence the binding of Ca^2+^ to zGCAPs and how Ca^2+^-induced conformational transitions in zGCAPs are influenced.

Our results indicate that myristoylation has a strong impact on the regulatory properties of two zGCAPs (2 and 5), but it does not facilitate Ca^2+^-dependent membrane binding for all zGCAPs. Further Mg^2+^ ions control the Ca^2+^-affinity as well as the Ca^2+^-induced conformational changes in zGCAPs.

## Results

### Acylation of zGCAPs in living cells

**Figure 1:**
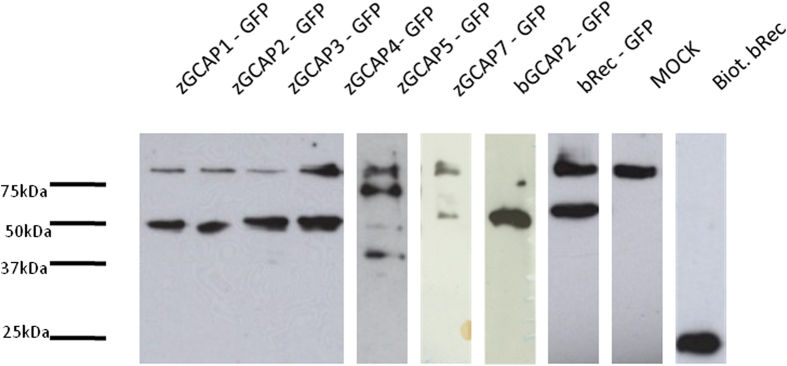
Visualization of acyl incorporation in various NCS proteins in mammalian cell lysates. HEK 293 cells were transfected with GFP constructs of NCS proteins and supplemented with azido-dodecyl acid and the biotin alkyne derivative was added to the cell lysate. Samples were electrophoresed and electrotransferred to a blot foil. The blot was developed by incubation with peroxidase coupled streptavidin and exposed to an ECL film. Total amount of protein in cell lysates that were loaded per lane was between 7 and 25 μg.

Green-fluorescent protein (GFP) constructs of NCS proteins including all zGCAPs, bovine GCAP2 and recoverin were used to transfect HEK 293 cells, which were also supplemented with azido-dodecyl acid (a myristoyl substitute). This allowed us to incorporate an acyl moiety into an NCS protein in a living cell. Successful attachment of the acyl group was monitored in a subsequent copper (I)-catalyzed azide-alkyne cycloaddition using a biotin alkyne derivative forming a triazole ring. Thus, NCS proteins that were labeled with biotin could be detected via streptavidin coupled to peroxidase. Each transfected cell sample was analyzed by sodium dodecyl-sulfate polyacrylamide gel electrophoresis, blot transfer and presence of biotin in the protein band was visualized by peroxidase staining (Fig. 1). Since GFP fusion constructs of NCS proteins were used we expected labeling of bands around 50 kDa, which was observed for all NCS proteins (Fig. 1). Most intense staining was detected for GFP fusion proteins of bovine GCAP2, recoverin and zGCAP1, 2, 3 and 4. Weaker staining was seen for zGCAP5 and zGCAP7. Despite differences in protein loading, these results indicated also different expression levels after transfection. The band above 75 kDa visible in all lanes except in that of biotinylated recoverin resulted from a labeled protein that was already modified in Mock transfected control cells (Fig. 1, note that the corresponding band in bGCAP2-GFP is only partially visible). Purified recombinant wildtype recoverin without a fusion part was biotinylated via covalent linkage to a primary amine and served as a positive control. It was intensely labeled (Fig. 1). These results showed that all zGCAPs (as well as bovine GCAP2 and recoverin) were posttranslational acylated in living cells.

**Figure 2:**
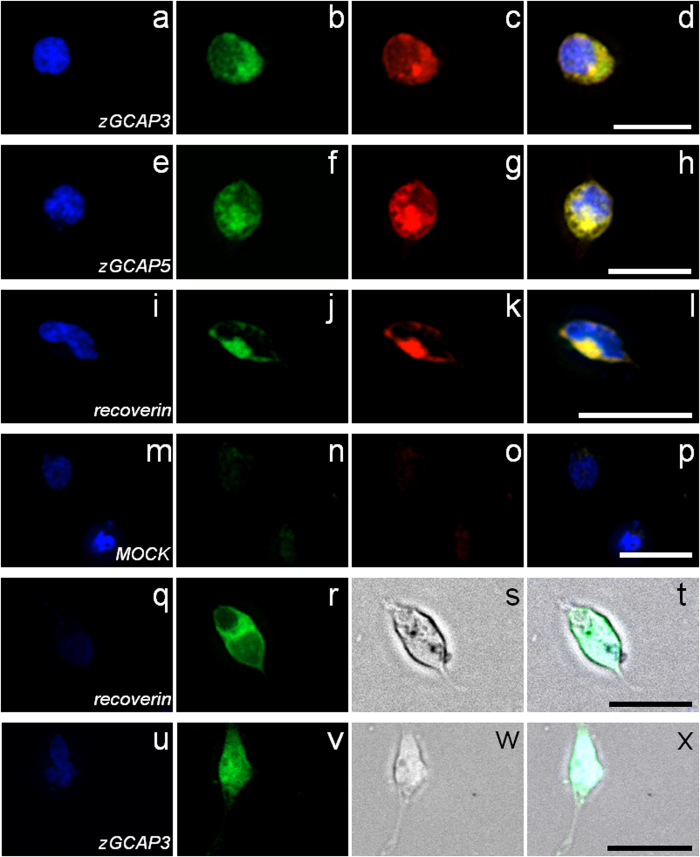
Acylation of zGCAPs in living cells by double fluorescence detection. HEK 293 cells were transfected with GFP constructs of zGCAPs and recoverin, which were acylated *in vivo* and labeled with the DIBO-TAMRA dye. Nuclear DAPI staining is displayed in left most column (**a,e,i,m,q,u**), GFP fluorescence in second left column (**b,f,j,n,r,v**), DIBO-TAMRA fluorescence in the third column (**c,g,k,o**) and an overlay of all signals in fourth column (**d,h,l,p,t,x**). GFP and DIBO-TAMRA fluorescence mainly overlaps for cytoplasmic regions. Pictures in panels s and w (column three) are bright field images of HEK cells that were not treated with the azido myristic acid substitute. Mock transfected cells are shown as controls. Figures were taken by using the LUCP PlanFi 40 x/0.60 olympus objective and the DAPI/Fitc/TexasRed Filter Set (Olympus). Scale bars: 20 μm.

In a second alternative approach we employed a copperless cycloaddition suitable for introducing a fluorescent dye (DIBO-TAMRA-dye) to the fatty acyl group of the NCS proteins in living cells. Thus we were able to colocalize the putative *N*-terminal attached fatty acyl chain and the *C*-terminal attached GFP to the protein in transfected cells. Figure 2 gives an overview of the results obtained with fluorescence microscopy for recoverin, zGCAP3 and 5. GFP and TAMRA fluorescence mainly overlapped for cytoplasmic regions indicating the presence of a covalently attached acylgroup on fluorescently labeled zGCAP3 and 5 (Fig. 2a–h). A similar localization pattern was observed with the other zGCAPs (data not shown). Cells expressing GFP-labelled zGCAP3 and recoverin (Fig. 2q–x) that were not modified by the azido-modified myristic acid substitute appear normal in shape comparable to previous results obtained with mammalian GFP-labelled GCAPs^19^,^20^. However, we observed no uniform spreading to the nucleus. Further, when we added the myristic acid substitute allowing the subsequent cycloaddition with the DIBO-TAMRA dye cell shape was affected leading to the round form visible in Fig. 2a–l (see also Figure S1 in supplement). Myristoylated recoverin was found in the vicinity to membranes, but it was also detected in restricted cytosolic regions (Fig. 2j–l and r–t). This might indicate partial association of recoverin with membrane structures at low Ca^2+^-concentration, which had been observed in previous studies and is mainly due to hydrophobic/ electrostatic interactions^21^,^22^,^23^.

### Do zGCAPs perform a Ca^2+^-myristoyl switch?

**Figure 3:**
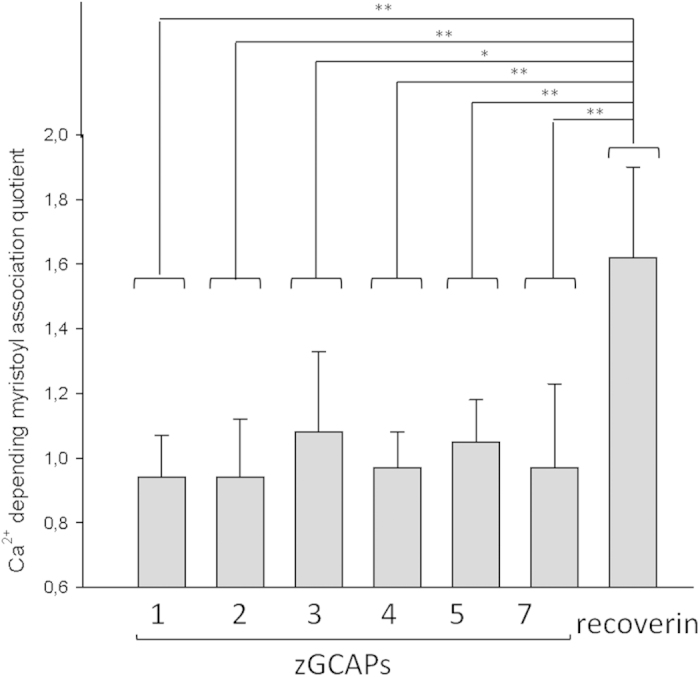
Association of zGCAPs with membranes. zGCAPs were incubated with ROS membranes in the presence and absence of Ca^2+^ (10 mM EGTA or 3 mM CaCl_2_, respectively) and the amount of attached proteins after equilibrium centrifugation was determined by densitometry. The ratio of Ca^2+^-saturated versus Ca^2+^-free zGCAP is displayed and compared with recoverin as positive control. Values ± SD are from 3–7 independent determinations. Statistical analysis was done by applying a Student’s t-test.

Our results showed that zGCAPs are modified by acylation (in the experiments above by a pseudo-myristoylation), which was tested in living and in disrupted cells. This prompted us to ask, whether zGCAPs can perform a Ca^2+^-myristoyl switch, which is typically observed in other NCS proteins like recoverin, neurocalcin δ, VILIP or hippocalcin^24^,^25^,^26^,^27^. In order to obtain a quantitative assessment on this topic we performed an equilibrium centrifugation assay. For this purpose we co-expressed zGCAPs and recoverin with yeast NMT in *E.coli* and purified them afterwards. Principal attachment of the myristoyl group was verified by a click chemistry reaction involving azido-dodecanoic acid and the alkyne derivative of biotin as described above. We then incubated myristoylated zGCAPs and recoverin with isolated photoreceptor outer segment membranes in the absence and presence of Ca^2+^ (Fig. 3). No zGCAP form showed a significant difference in binding to membranes under these conditions. Thus, no zGCAP performed a Ca^2+^-myristoyl switch thereby confirming our previous results that we reported for zGCAP3 and 4 resembling those obtained earlier with mammalian GCAP1 and GCAP2^28^,^29^. Recoverin as the prototype of a Ca^2+^-myristoyl switching NCS protein served as positive control (Fig. 3).

### Target regulation by zGCAPs

Nonmyristoylated zGCAPs exhibit different activity profiles when targeting membrane bound GCs^12^. We compared previously published data on nonmyristoylated zGCAPs with those obtained with myristoylated zGCAPs in the present study. A characteristic parameter for activity regulation is the [Ca^2+^] at which the GC activity in the presence of a GCAP molecule is halfmaximal denoted as IC_50_ value and listed in Table 1. Interestingly, large differences (> 2-fold) were only visible for zGCAP2 and zGCAP5. With a myristoyl group attached the Ca^2+^-sensitivity decreased about 4.7-fold for zGCAP2 and increased 5.7-fold for zGCAP5.

### Ca^2+^-binding to myristoylated zGCAPs

**Figure 4:**
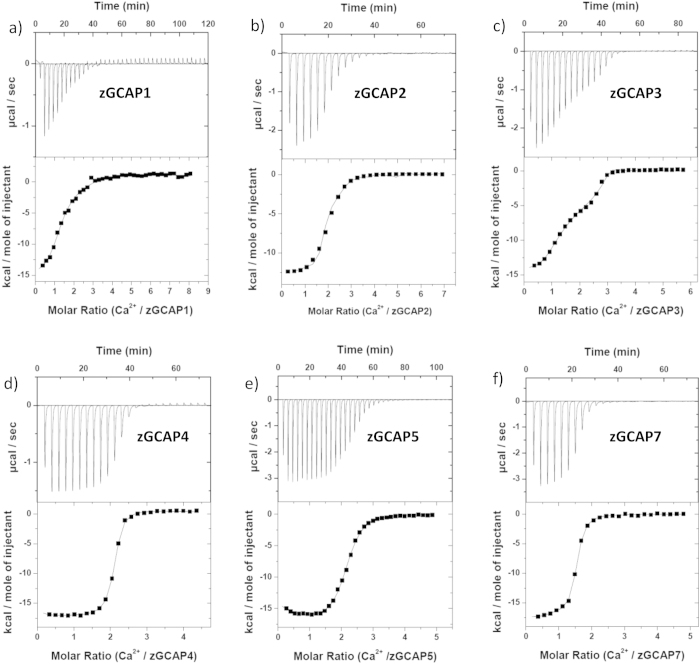
Ca^2+^-binding to myristoylated zGCAP-isoforms. Representative ITC measurements with 10.2 μM zGCAP1 (**a**), 12.8 μM zGCAP2 (**b**), 18.7 μM zGCAP3 **(c**), 10.2 μM zGCAP4 (**d**), 19.5 μM zGCAP5 (**e**), 18.5 μM zGCAP7 (**f**). The upper part shows the heat pulse for every injection, the lower part shows the corresponding molar enthalpy change. Data analysis by curve fitting to three or two Ca^2+^ binding sites yielded dissociations constants (K_D_^app^) and and enthalpy changes (ΔH) given in Table 2.

**Figure 5:**
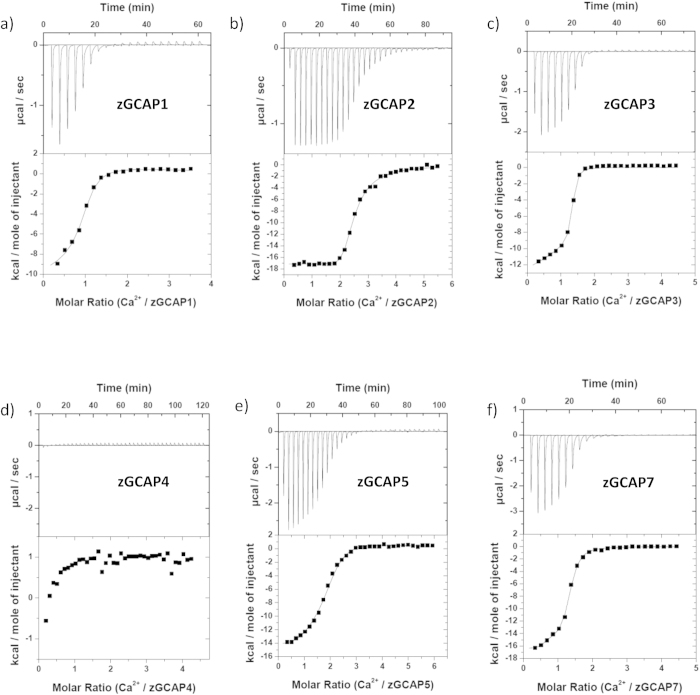
Ca^2+^-binding in the presence of 1 mM Mg^2+^ to zGCAP-isoforms. Representative ITC measurements with 10.2 μM zGCAP1 (**a**), 10.2 μM zGCAP2 (**b**), 16.7 μM zGCAP3 (**c**), 12.5 μM zGCAP4 (**d**), 18.3 μM zGCAP5 (**e**), 19.7 μM zGCAP7 (**f**). The upper part shows the heat pulse for every injection, the lower part shows the corresponding molar enthalpy change. Data analysis by curve fitting to three or two Ca^2+^ binding sites yielded dissociations constants (K_D_^app^) ^a^nd and enthalpy changes (ΔH) given in Table 2.

All zGCAPs contain four EF-hand motifs in their primary structure, where the first one is suggested to bind no Ca^2+^ under physiological conditions as it was observed for mammalian GCAP1 and 2^8^. So far no precise values of Ca^2+^-binding to myristoylated zGCAPs are available and furthermore it is not known, whether Ca^2+^-binding is affected by physiological concentrations of Mg^2+^. We used a calorimetric approach (ITC) to analyze the energetics of Ca^2+^-binding to zGCAPs in the presence and absence of 1 mM Mg^2+^, which allows us to determine precise values of apparent dissociation constants (K_D_) for multiple binding sites and the associated changes in binding enthalpy (ΔH). For each titration Ca^2+^-free zGCAP was kept in a temperature controlled compartment, in which a series of small volumes of CaCl_2_ was injected. Representative examples for all zGCAPs are shown in Figs 4,5. Heat pulses were in almost all cases exothermic with a maximum heat release between −14 kcal per mol of CaCl_2_ (zGCAP3) and −17 kcal per mol of CaCl_2_ (zGCAP7). Small endothermic responses were only observed with zGCAP1 (Figs 4a,5a). However, despite numerous repetitions we could not get reproducible results for zGCAP1. Only once we detected a response pattern as seen in Fig. 4a. In the presence of Mg^2+^ only two titrations out of six were successful (Fig. 5a). We interpret these findings with the tendency of some NCS proteins to form higher order oligomers, a phenomenon we have previously observed^30^. Data could be fit by a sequential three site binding model yielding three distinct K_D_ values except for zGCAP7, where only a two site model was applicable (Table 2). In all zGCAPs at least two binding sites exhibited high affinities for Ca^2+^ with K_D_ values in the submicromolar range (K_D_^1^ and K_D_^2^ in Table 2), for zGCAP3 also the third site (K_D_^3^) showed high affinity for Ca^2+^, whereas for other zGCAPs the affinity of K_D_^3^ was in the micromolar range.

In the presence of 1 mM Mg^2+^the multiphasic binding isotherm for Ca^2+^-binding gave a best fit with a two site model for zGCAP1, 2, and 3. This result can be best interpreted as having two high affinity sites filled with Ca^2+^, but the lower affinity site being occupied by Mg^2+^ (Fig. 5 and Table 2). However, zGCAP5 and 7 showed a somewhat different behavior: the binding isotherm of zGCAP5 still obeyed to a sequential three site model, but all three K_D_ values were shifted to lower affinity (Table 2). In contrast, zGCAP7 displayed almost equal values for K_D_^1^ and K_D_^2^ indicating no binding of Mg^2+^ to either of these binding sites.

Surprisingly, we were unable to record any response with zGCAP4 in the presence of Mg^2+^ (Fig. 5d), although reliable binding isotherms were recorded without Mg^2+^ (Fig. 4d and Table 2).

Size exclusion chromatography of myristoylated zGCAPs revealed that the monomeric form was the dominant species for all zGCAPs except for zGCAP5 and 7. A Ca^2+^-dependent shift in the monomer-dimer equilibrium was only observed for zGCAP2, but the monomeric zGCAP was with approx. 80% the dominant form. High molecular mass oligomers were not detected except occasionally for zGCAP7 and to a lower extent for zGCAP5 (data not shown).

### Ca^2+^-induced conformational changes of myristoylated zGCAPs

**Figure 6:**
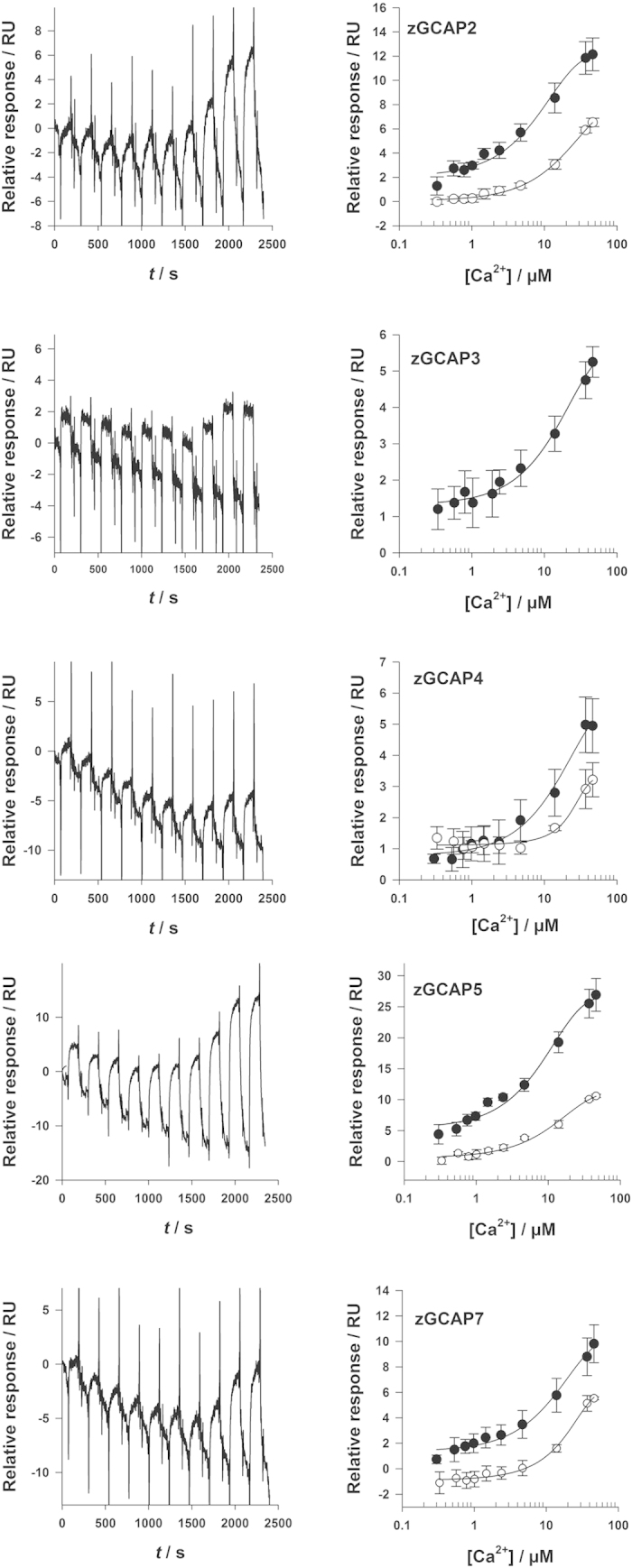
SPR responses of myristoylated zGCAP isoforms at increasing free [Ca^2+^]. Examples of original SPR recordings are shown in the left panels, evaluation of data without Mg^2+^ (•) and with 1 mM Mg^2+^ (O) are shown on the right panels. Data fitting was obtained by a sigmoidal Hill curve resulting in K_1/2_^SPR^ values listed in Table 3.

The lack of a Ca^2+^-myristoyl switch operation in zGCAPs (Fig. 3) led us suggest that Ca^2+^-induced conformational changes in zGCAPs might be less pronounced than in recoverin that undergoes a large rearrangement of its three-dimensional fold during the exposure of its myristoyl chain^24^. We recently developed a technique to detect subtle changes in protein conformation during conformational transitions of Ca^2+^-sensors by applying SPR spectroscopy^30^,^31^,^32^. The technique allowed us to correlate even small changes in conformation with both a rearrangement of the protein hydration shell and protein hydrodynamic properties. Thus, we applied this technique for the detection of conformational transitions in zGCAPs in the absence and presence of Mg^2+^. For this purpose zGCAPs were immobilized at high density on a dextran-coated sensor chip surface and pulses of CaCl_2_ were injected into the flow cell system of the SPR device resulting in a pattern of increasing amplitudes, when the [Ca^2+^] is increased (Fig. 6). Evaluation of the titration revealed a K_1/2_^SPR^ in the micromolar range (Table 3) as previously determined and discussed for mammalian GCAPs, recoverin and other Ca^2+^-sensors^30^,^31^,^32^. Performing the same titration series in the presence of 1 mM Mg^2+^(Fig. 6, open circles) shifted the K_1/2_^SPR^ to higher values, in particular for zGCAP2 (Table 3). However, we lack data for zGCAP3, since flushing a zGCAP3-coated surface with Mg^2+^-containing buffer diminished any response during titration. Apparently, Mg^2+^ increased the instability of zGCAP3 on the chip surface.

Finally, the maximal amplitudes that were reached at the end of the Ca^2+^-titration (in the absence of Mg^2+^) differed significantly among the proteins exhibiting the following order: zGCAP5 > zGCAP2 > zGCAP7 > zGCAP3 = zGCAP4. No signals were observed for zGCAP1, although the purified protein was functional shown by the GC activation assay.

We conclude from these results that all zGCAPs undergo Ca^2+^-induced conformational changes, which however have different consequences for the protein hydrodynamic properties indicating differences in the extent of conformational transitions.

## Discussion

Calcium sensor proteins like the group of NCS proteins are involved in unique patterns of cellular regulatory pathways and therefore mediate various physiological responses including ion channel function, enzyme activity control and cellular trafficking^2^,^3^. NCS proteins are able to specifically recognize their targets despite the fact that they share a high degree of amino acid sequence homology and that the overall three-dimensional folding is nearly identical in the core regions (e.g. sequential order of EF-hand structures), at least in those NCS proteins, of which the tertiary structure is known (a comparative summary is given in ref. 33). Thus, it is a fundamental question how specific target recognition processes are achieved. Moreover, a related central question is how differences in Ca^2+^-signaling that result in specific cellular responses can be mediated by rather similar Ca^2+^-sensor proteins, which are very often expressed in the same cell type. In order to work on these issues we chose to compare zGCAPs representing one subfamily of NCS proteins. These NCS proteins are well suited for a comparative analysis, since previous work has shown 1) that all zGCAPs are expressed in photoreceptor cells in the larval and adult stages of the zebrafish retina^13^,^14^,^15^,^18^, 2) that all forms are functional Ca^2+^-binding proteins^12^,^15^,^18^ and 3) that all forms can control the activity of membrane bound sensory guanylate cyclases in a Ca^2+^-dependent manner^12^,^13^,^15^,^16^,^18^.

Amino-terminal myristoylation has a strong impact on the Ca^2+^-sensitivity of mammalian GCAP1, but less influence on the regulatory properties of mammalian GCAP2^10^,^29^,^34^. However, for most zGCAPs it has not been clarified, whether they are myristoylated in living cells or under *in vitro* conditions. We here demonstrate using complementary approaches that the six zGCAPs are myristoylated in cell lysates and in living cells. Further, myristoylation is not involved in reversible Ca^2+^-dependent membrane association (Figs 2,3). Instead, myristoylation of zGCAPs enables a differential response pattern of Ca^2+^-signaling as outlined below.

Our previous work on the six nonmyristoylated zGCAPs revealed that zGCAP1, 2 and 3 display GC-activating response curves that are halfmaximal around 30 nM free [Ca^2+^] and curves of zGCAP4, 5 and 7 are halfmaximal around 400 nM [Ca^2+^]^12^. Interestingly, attaching a myristoyl group changed the Ca^2+^-sensitive response curves for two zGCAPs (2 and 5) leading to one group of zGCAPs with high Ca^2+^-sensitivity (zGCAPs 1, 3 and 5) and one with low Ca^2+^-sensitivities (zGCAPs 2, 4 and 7). Myristoylation is catalyzed by a retinal NMT, which is not active before 7 dpf due the myristoylation pattern we reported previously for zGCAP3^15^. Thus, myristoylation appears as a final adjustment being necessary for fine-tuning of zGCAP function, but it is not necessary for their general function^15^.

The Ca^2+^-sensing properties of myristoylated zGCAPs were broadly consistent with the primary sequences that contain three predicted functional EF-hand Ca^2+^-binding sites as described above and presented in Table 2. Further, ITC data showed that Mg^2+^ can bind to zGCAP1, 2 and 3 by occupying one EF-hand and leaving the other two for binding Ca^2+^. Indirect evidence of Mg^2+^-binding was obtained for zGCAP5, in which the low affinity of one Ca^2+^-binding site decreased by the presence of Mg^2+^ (Table 2) indicating competition with Ca^2+^. ITC measurements with zGCAP4 were only possible in the absence of Mg^2+^, since the isolated protein aggregated during the time course of an experiment. The only exception in this NCS protein group was zGCAP7: fitting ITC data to a two site-binding model was sufficient for Ca^2+^-titrations in the absence and presence of Mg^2+^. This could mean that Mg^2+^can occupy one of the remaining (Ca^2+^-free) EF-hands without affecting Ca^2+^-binding or that Mg^2+^ does not bind to zGCAP7.

Amino acid sequence comparison of the canonical EF-hands (2, 3 and 4) in all zGCAPs revealed that EF-hand 4 exhibits the highest degree of conservation and identity in the EF-loop positions, in which the coordinating ligands are denoted *X,Y,Z-Y-X,-Z*^35^. Less sequence homology is seen in EF-hands 2 and 3. For example zGCAP5 harbors an uncommon Cys at the position *–Y* (EF-hand 2 and 3), a position that is known to provide an invariant oxygen ligand for coordinating Ca^2+^ or Mg^2+^. Val is present in zGCAP7 at *−Y* (EF-hand 2) and it does also not belong to the amino acids that are frequently observed in EF-hands in this position^35^. The same position *–Y* is also peculiar in EF-hand 3 that contains a Lys (common) in zGCAP3, 4 and 7, but again an uncommon Cys in zGCAP1, 2 and 5. These amino acid substitutions in EF-hands 2 and 3 could therefore be the molecular determinants for tuning the different Ca^2+^ affinities.

Ca^2+^-induced conformational changes in zGCAPs are thought to trigger target activation and are therefore key control steps in Ca^2+^-mediated feedback loops. Probing these changes by a recently established surface plasmon resonance approach (Fig. 6 and Table 3) we observed distinct changes in the hydrodynamic properties of zGCAPs triggered by Ca^2+^. The K_1/2_^SPR^ value that is estimated from these titrations is an empirical parameter, which describes a concerted action of a binding step involving a conformational switch^30^,^31^,^32^. Therefore, the apparent dissociation constants listed in Table 2 are a different set of parameters, which are not identical to the K_1/2_^SPR^ values. Instead, the K_1/2_^SPR^ values might reflect a major structural reorganization in zGCAPs as we observed and discussed for mammalian GCAPs and recoverin before^30^,^31^,^32^. For isoforms zGCAP2, 4, 5 and 7 we determined K_1/2_^SPR^ values in the lower micromolar range matching the low affinity Ca^2+^-binding site in zGCAP2 and zGCAP5 and to some extent also in zGCAP4.

In contrast, we measured for zGCAP3 and zGCAP7 only three or two high affinity Ca^2+^-binding sites and the meaning of the K_1/2_^SPR^values is less obvious for these proteins. At the moment we can only speculate that the rearrangement of the protein hydration shell, which is reflected and measured by our SPR approach, is differently affected in zGCAP3 and 7 indicating different surface properties. For example, a Ca^2+^-dependent or Ca^2+^-independent monomer-dimer equilibrium like it is observed for mammalian GCAP2 or GCAP1, respectively^36^,^37^,^38^,^39^, could have an impact on the formation of the hydration shell around zGCAP3 or 7. Interestingly, zGCAP7 undergoes a shift in monomer-dimer rearrangement^12^, when [Ca^2+^] is changed, an observation that might also account for other peculiarities of zGCAP7 (e.g. two Ca^2+^-binding sites instead of three, no apparent effect of Mg^2+^).

Presence of Mg^2+^ caused less than two-fold shifts in K_1/2_^SPR^ values (Table 3), but also a distinct decrease in response amplitudes (Fig. 6, right panels). Only the K_1/2_^SPR^ of zGCAP2 was more affected and shifted by a factor of 2.4. We made similar observations recently on mammalian GCAP1 and on retinal disease related mutants of GCAP1^30^. Collectively, these results indicated that the hydrodynamic properties of zGCAPs are more influenced by Ca^2+^ than by Mg^2+^-binding. Recent molecular dynamics simulations further showed that mammalian GCAP1 in its Mg^2+^-bound forms has a less solvent exposed surface than the Ca^2+^-saturated GCAP1 state^40^. This finding would be consistent with the smaller response amplitudes observed for zGCAPs in the presence of Mg^2+^, which mirror changes of hydrodynamic diameters and of the rearrangement of the water-protein interface.

## Methods

### Cloning of zGCAPs and GFP-zGCAP constructs

For heterologous expression in *E.coli* the coding sequences of all zGCAP forms were amplified by PCR and ligated into a pET21-vector earlier^12^,^15^. In order to obtain *N*-terminal acylated zGCAPs in *E. coli*, point mutation of zGCAP1, 4 and 5 was necessary to create a consensus sequence for yeast NMT. The cloning of the point mutants zGCAP4-A^6^S and zGCAP5-D^3^N were described before^12^,^15^,^18^. Accordingly, we prepared a zGCAP1-G^6^S-mutant employing primers 5′-AAACATATGGGCAATTCAACGAGCAGC-3′ and 5′-AACGAATTCTTAAACGCTGTGTCTCCGGTTATG-3′.

ZGCAP-GFP-fusions were obtained by PCR on wildtype sequences adding restriction sites (*NheI* and *XhoI*) and a Kozak sequence in front of the start codon and removing the stop codon. Primer sequences are given in the supplement. The amplified PCR products were ligated into the pTurboGFP-N vector (Evrogen) according to standard protocols. Sequences of all zGCAPs were verified by DNA sequencing.

### Culture and transfection of HEK 293 cells

HEK-293 cells were cultivated in Dulbecco’s modified Eagle’s medium, pH 7.4 (DMEM and GlutaMaxTM-I, Gibco®) including 10% (v/v) fetal bovine serum (Gibco®) and antibiotics/ antimycotic (Gibco®) at 37 °C and 5% CO_2_. Cells were transfected with the corresponding plasmid DNA of NCS proteins (GCAPs and recoverin) or their GFP fusion constructs. Transfection was performed by electroporation essentially as described before^41^. Briefly, the transfection was tested with different DNA concentrations of 5–50 μg at a cell density of 9 × 10^6^ for obtaining high transfection rates. After electroporation the mixture was spread on a 6 well (2.5 ml DMEM media) or 24 well plate (325 μl DMEM media) and incubated for 1 to 2 days at 37 °C. Alternative transfection was performed by lipofection using the PolyFect reagent exactly according to the protocol of the manufacturer (Quiagen, Venlo, Netherlands)

For *in vitro* analysis cells were centrifuged for 5 min at 1000 × g, the pellet was stored at –80 °C or immediately used for further experiments.

### Post-translational modification of zGCAPs by fatty acylation detetcted by copper-free cycloaddition (Click chemistry) in living cells

Acylation of heterologous expressed zGCAPs was detected in living cells and in lysed cell preparations. In both cases a 12-azido-dodecanoic acid was used as a myristic acid substitute. The labeling in living cells was done in adherent HEK-293 cells. Cover slips were inserted into one well of a 24 well chamber petri dish and coated with 0.1 mg/ml poly-L-lysine solution overnight to improve the adhesion of the HEK cells. After two washing steps with PBS (137 mM NaCl, 2.7 mM KCl, 12 mM phosphate, pH 7.4), the cells were seeded on the cover slips and incubated for 2–3 days. After one day of incubation 12-azido dodecanoic acid (40 μM) was added and cells were further incubated. Afterwards cells were washed twice with PBS before adding 1.25 μM of the alkyne-dye reagent DIBO-TAMRA (Invitrogen, Eugene, USA). Cells were washed four times to remove all non-bound dye residues and fixated with 4% formaldehyde (15 min, RT). To remove the fixation solution, two washing steps with PBS, one with TBST (155 mM NaCl, 20 mM Tris-HCl, pH 7.4; 0.05% (v/v) Tween-20), one with TBS and a final one with PBS followed before the cover slips were used on a microscopic slide for fluorescence microscopy. Bovine recoverin^3^ served as positive control.

### Detection of acylated zGCAPs in lysed cells using a Cu(I)-catalyzed Huisgen cycloaddition

About 2.25 × 10^6^ HEK-Cells were used in one transfection charge resulting in 10–30% successful transfection rate expressing zGCAPs with a *N*-terminal covalently attached 12-azido-dodecanoic acid. Cells were disrupted in 200 μl lysis buffer (1% (w/w) SDS, 5U DNAse, mammalian protease inhibitor cocktail from Sigma containing AEBSF (104 mM), Aprotinin (80 μM), Bestatin (4 mM), E-64 (1.4 mM), Leupeptin (2 mM), Pepstain A (1.5 mM), 50 mM Tris-HCl, pH 8.0) and incubated for 30 minutes on ice. The cell suspension was centrifuged (13,000–18,000×g; 4 °C; 5 min) and the resulting supernatant was transferred to a new reaction tube and shock frozen with liquid nitrogen. Samples were stored at −20 °C or used immediately.

To label the azido-dodecanoyl group in the NCS proteins we added to the respective lysed cell extract an alkyne derivative of biotin, which reacted in a Cu (I) catalyzed cycloaddition with the azido group, following the protocol of the Click iT^®^ Protein Reaction Buffer Kit (Invitrogen, Eugene, USA). Briefly, we used 60 μL of the lysed cell extract and added ten μl 40 μM CuSO_4_ (final concentration of 2 μM). The reaction was allowed to proceed for 20 min in a test tube that rotated end-over-end. Subsequently, the suspension was filled into a concentrating device (3K; Amicon® Ultra; Millipore, Ireland) and centrifuged (15000 × g, 30 min, 18 °C). Samples were run on a SDS polyacrylamide gel, blotted and the presence of the biotin-acyl group was tested by incubating the blot with peroxidase-coupled streptavidin for one hour. Acylated protein bands were visualized by the ECL system.

### Fluorescence microscopy

The microscopic analysis was performed on an Olympus IX81microscope with a LUCPPlanFi 40*/0.60 Olympus objective. For the detection of the TAMRA labeled azido-dodecanoyl moiety we used the Texas Red filter (excitation 568 nm). The GCAP-GFP constructs were detected with the FITC filter (excitation 488 nm) and the DAPI nuclei staining with the DPI filter (excitation 358 nm), respectively.

### Protein expression, purification and characterization

For biophysical characterization of zGCAPs proteins were overexpressed in BL21 *E.coli* cells as described previously^12^,^15^,^18^. To obtain myristoylated zGCAPs, *E.coli* cells were cotransformed with the plasmid pBB131 containing a gene for the yeast (*S. cerevisiae*) NMT. A consensus sequence for yeast NMT is present in zGCAP 2, 3 and 7. For zGCAP1, 4 and 5 we used the point mutations described above.

After cell lysis zGCAP 3 and 4 were isolated from the soluble fraction, but zGCAP1, 2, 5 and 7 were extracted from the insoluble fraction by homogenization in 6 M guanidinium hydrochloride and dialytic refolding against Tris-buffer (20 mM Tris-HCl, 150 mM NaCl, 1 mM DTT pH 7.5). After one buffer change insoluble material was removed by centrifugation (100,000 × g for 30 min). The purification was a combination of size exclusion chromatography (SEC) and anion-exchange chromatography (AEC). Prior to chromatography steps the volume of zGCAP solutions was reduced by ammonium sulfate precipitation. Afterwards, zGCAPs were resolved in Tris-buffer and were applied onto the SEC column (Superdex 75,GE Healthcare, Germany) in the presence of either 2 mM EGTA (zGCAP1, 3, 4) or 2 mM CaCl_2_ (zGCAP2, 5, 7). Fractions containing zGCAPs were further purified using an AEC column (HiLoad 26/10 Q Sepharose; GE Healthcare, Germany) equilibrated in Tris-buffer with 2 mM EGTA. Chromatography was performed with a gradient of 200–550 mM NaCl in 70 mL. Analytical size exclusion chromatography was performed as described previously^12^,^18^,^30^ using a Primaide HPLC system (Hitachi VWR International GmbH, Darmstadt, Germany),

Analysis of purified zGCAP samples by sodium dodecyl-sulfate polyacrylamide gel electrophoresis and determination of GC activity (three to five repetitions) in the presence of zGCAPs as a function of the free [Ca^2+^] was done exactly as described^12^,^15^,^18^. The membrane binding of myristoylated NCS proteins was tested with isolated rod outer segment membranes as described^15^,^18^.

### Isothermal titration calorimetry (ITC)

ITC experiments with zGCAP-isoforms were performed on a VP-ITC from MicroCal (Northhampton, MA) exactly as described previously for mammalian GCAP1 variants^42^. Briefly, purified zGCAP-isoforms were present in the recording cell in titration buffer (5`mM Tris/HCl, pH 7.5; 150 mM KCl) at 10–21 μM and were titrated with 3–5 μL of a 1–2 mM CaCl_2_ stock solution at T = 25 °C. The titration buffer was decalcified using a self-packed gravity flow Chelex 100 column (Bio-Rad). The remaining Ca^2+^concentration was determined by a BAPTA absorption assay and was found to range between 30 and 100 nM. All buffers were filtered (0.22 μm) and degassed at least 1h before use. At least three independent repetitions were made for each titration set, if not stated otherwise.

Reference injections of Ca^2+^ into decalcified buffer without any zGCAP was performed, but did not show significant heat changes in the recording cell. Each titration was analyzed by a model implemented in the software Origin (MicroCal) assuming either three or two Ca^2+^ binding sites. The best fitting results out of these models were used to obtain dissociation constants K_D_^app^ and enthalpy changes (ΔH).

### Surface plasmon resonance experiments

For SPR experiments, we used exactly the same decalcified buffer conditions as for the ITC experiments, except that Tween20 was added to a final concentration of 0.005% (v/v).The Ca^2+^-titration experiments and data analysis were performed as outlined elsewhere^30^,^31^,^32^ and repeated six to eight times, with immobilized zGCAP3 four times. In brief CaCl_2_ of the highest grade available was dissolved in the decalcified buffer to a final concentration of 46 mM. This stock solution was used to obtain Ca^2+^ samples of 0.4 μM, 0.7 μM, 0.9 μM, 1.1 μM, 1.6 μM, 2.5 μM, 4.8 μM, 14 μM, 37 μM and 46.2 μM. All buffers were filtered (0.22 μm) and degassed for at least 1h before use. Immobilization of protein samples was achieved by attaching them to the carboxy-methyl dextran matrix of CM5 sensorchips (GE Healthcare) via the terminal amino group or via internal accessible lysine residues. Typical immobilization densities ranged from 3.5 ng to 10.5 ng/mm^2^.

## Additional Information

**How to cite this article**: Sulmann, S. *et al.* Retina specific GCAPs in zebrafish acquire functional selectivity in Ca^2+^-sensing by myristoylation and Mg^2+^-binding. *Sci. Rep.* **5**, 11228; doi: 10.1038/srep11228 (2015).

## Supplementary Material

Supplementary Information

## Figures and Tables

**Table 1:** Activating properties of myristoylated and nonmyristoylated zGCAPs.

**zGCAP**	**1**	**2**	**3**	**4**	**5**	**7**
**IC**_**50**_ **myr (nM)**	58	163	30^a^	570^b^	77	145
**IC**_**50**_ **nonmyr (nM)**	30^c^	35^c^	25^c^	520^b^	440^c^	180^c^

IC_50_ value is defined as the [Ca^2+^] at which the GC activity in the presence of a GCAP molecule is halfmaximal.

^a^data taken from ref. 15

^b^data taken from ref. 18

^c^data taken from ref. 12.

**Table 2:** Thermodynamics of Ca^2+^ binding to zGCAP isoforms in the presence and absence of physiological Mg^2+^.

**zGCAP form**		**Sequential three site model**					
		**Dissociation constant K_D_ (μM)**			**Enthalphy change ∆H (kcal/mol)**		
		**K_D_^1^**	**K_D_^2^**	**K_D_^3^**	**∆H^1^**	**∆H^2^**	**∆H^3^**
**zGCAP1**^a^		0.19	5.26	284.9	–14.0	–9.4	+69.6
**zGCAP2**		0.021 ± 0.12	0.41 ± 0.26	6.46 ± 1.84	–16.8 ± 0.7	–16.5 ± 1.1	–2.6 ± 2.7
**zGCAP3**		0.014 ± 0.004	0.24 ± 0.09	0.30 ± 0.02	–13.0 ± 1.3	–9.6 ± 2.6	–2.1 ± 1.1
**zGCAP4**		0.033 ± 0.38	0.068 ± 0.037	154.5 ± 134.5	–18.8 ± 2.2	–15.4 ± 1.4	–2.0 ± 4.3
**zGCAP5**		0.37 ± 0.19	0.61 ± 0.10	2.91 ± 2.45	–19.5 ± 1.7	–12.1 ± 1.2	–0.5 ± 0.4
	Mg^2+^	0.91 ± 0.074	1.94 ± 0.29	47.05 ± 39.85	–14.2 ± 0.9	–12.5 ± 1.6	–0.6 ± 0.2
		**Two site model**					
		**K**_**D**_^**1**^	**N**^**1**^	**K**_**D**_^**2**^	**N**^**2**^	**∆H**^**1**^	**∆H**^**2**^
**zGCAP1**^a^	Mg^2+^	0.065 ± 0.022	0.61± 0.23	0.35 ± 0.14	1.12 ± 0.4	–12.8 ± 2.0	–1.5 ± 1.4
**zGCAP2**	Mg^2+^	0.012 ± 0.005	0.89 ± 0.08	1.64 ± 0.9	1.88 ± 0.5	–17.3 ± 0.01	–6.4 ± 1.0
**zGCAP3**	Mg^2+^	0.053 ± 0.0045	1.10± 0.01	0.093 ± 0.005	0.64 ± 0.1	–15.2 ± 1.0	–3.2 ± 0.7
**zGCAP7**		0.005 ± 0.004	0.61 ± 0.10	0.28 ± 0.01	0.89 ± 0.04	–17.3 ± 0.1	–16.8 ± 0.2
	Mg^2+^	0.003 ± 0.002	0.51 ± 0.02	0.49 ± 0.03	0.80 ± 0.06	–16.5 ± 0.2	–15.4 ± 0.2

^a^Titration of GCAP1 with Ca^2+^ showed also small endothermic responses, in the absence of Mg^2+^ only one set of data was reliable (see main text).

**Table 3:** Halfmaximal SPR response value K_1/2_^SPR^ in the presence and in the absence of Mg^2+^.

**GCAP-Isoform (myristoylated)**	**K_1/2_^SPR^(μM)**	**K_1/2_^SPR^(μM)(+ 1 mM Mg^2+^)**
**zGCAP1**	N.D.	N.D.
**zGCAP2**	7.7 ± 0.5	18.7 ± 0.3
**zGCAP3**	40.3 ± 1.3	N.D.
**zGCAP4**	16.3 ± 0.6	24.1 ± 0.4
**zGCAP5**	8.3 ± 0.4	13.0 ± 0.4
**zGCAP7**	14.7 ± 0.6	20.9 ± 0.4
