# Erratum: Retina specific GCAPs in zebrafish acquire functional selectivity in Ca^2+^-sensing by myristoylation and Mg^2+^-binding

**DOI:** 10.1038/srep13508

**Published:** 2015-09-02

**Authors:** Stefan Sulmann, Farina Vocke, Alexander Scholten, Karl-Wilhelm Koch

Scientific Reports 5: Article number: 1122810.1038/srep11228; published online: 06102015; updated: 09022015.

In the Supplementary Information file originally published with this Article, the primer sequences for cloning the constructs were omitted. This error has now been corrected in the Supplementary Information that now accompanies the Article.

