# Supplementary material for: Retina specific GCAPs in zebrafish acquire functional selectivity in Ca2+-sensing by myristoylation and Mg2+-binding
Source: Sci Rep. 2015 Jun 10;5:11228. doi: 10.1038/srep11228 (PMC4462140; doi:10.1038/srep11228)
Supplement: Supplementary Information [file srep11228-s1.doc]

**Supporting information**

**Retina specific GCAPs in zebrafish acquire functional selectivity in Ca^2+^-sensing by myristoylation and Mg^2+^-binding**

Stefan Sulmann*, Farina Vocke*, Alexander Scholten and Karl-Wilhelm Koch^1^

Department of Neurosciences, Biochemistry Group, University of Oldenburg, D-26111-Oldenburg, Germany

*These authors contributed equally to this work


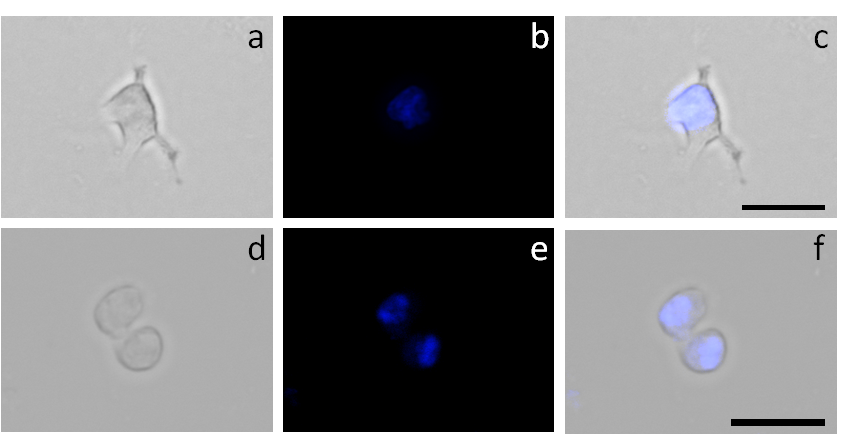


Figure S1: Effect of 12-azido-dodecanoic acid on HEK 293 cell shape. The upper part shows non-treated HEK 293 cells, the lower part shows cell with a more rounded appearance after incubation with 12-azido-dodecanoic acid (a and d, bright field image). DAPI staining is shown in b and e, overlays are displayed in d and f. Scale bar: 20 µm.
